# Emergence of an Israel faith-based community organization facilitating live donor kidney transplantation

**DOI:** 10.1186/s12882-018-0923-4

**Published:** 2018-06-07

**Authors:** Walter G. Wasser, Geoffrey Boner, Meni Koslowsky, Adi Lazar

**Affiliations:** 1 Division of Nephrology, Mayanei HaYeshua Medical Center, 51544 Bnei Brak, Israel; 2 Rambam Health Care Campus, 3109601 Haifa, Israel; 3 Department of Medicine, Tel Aviv University Sackler Faculty of Medicine (retired), Tel Aviv University, Ramat Aviv, 6997801 Tel Aviv, Israel; 4 Departments of Psychology, Bar-Ilan, 52900 Ramat Gan, Israel; 5 Ariel University, 44837 Ariel, Israel; 6 Department of Economics, Ariel University, 44837 Ariel, Israel

**Keywords:** Altruism, Kidney transplant, Community organization

## Abstract

**Background:**

The 2014 Consensus Conference on Best Practices in Living Kidney Donations recognized live donor kidney transplantation as the best treatment for late-stage kidney disease, yielding superior graft and patient survival, improved quality of life, fewer requirements for dialysis and increased cost-effectiveness compared to deceased donor kidney transplantation. Yet in spite of the excellent results of living kidney donation, the annual number of living kidney donors is declining in many countries, including the United States. In Israel, a non-profit organization, Matnat Chaim (“Gift of Life” in Hebrew), a faith-based initiative, has emerged as a major force for arranging living donor kidney transplantation mainly by facilitating altruistic living unrelated donor transplantation.

**Methods:**

A retrospective review of the records of live kidney donations facilitated by the Matnat Chaim organization and referred to Israel transplant centers, since the organization’s inception in 2009, was performed and compared to published data from the Israel Ministry of Health.

**Results:**

Matnat Chaim has facilitated 494 live kidney donations since its founding in February 2009 until the end of 2017. Of the 124 live kidney transplants performed in 2016, 111 (90%) were shown to be altruistic and unrelated. This large number of donations was associated with a doubling of the total number of kidney transplantations, performed in Israel (data published by the Israel Ministry of Health).

**Conclusions:**

The success of an Israel community organization in the promotion of kidney transplantation may serve as a model for other religious and non-religious communities worldwide.

## Background

### Kidney transplantation: The most effective form of kidney replacement therapy worldwide

Kidney transplantation results in both an increase in life expectancy and quality of life, as compared to individuals treated with dialysis [1–3]. Kidneys from deceased donors are the major source of organs for kidney transplantation worldwide [4, 5]. However, only a few countries have been able to obtain sufficient numbers of deceased donor organs to supply the needs of individuals waiting for kidney transplantation [6]. This has resulted in potential recipients having to remain on dialysis for a prolonged period, with many patients dying before receiving a graft. Initially, kidneys from living donors were only used when the donor was related to the recipient. Since the early 1970’s, advances in immunosuppression have resulted in kidney survival rates from non-related donors, which are similar to those obtained using organs from related living donors [6]. This has led to an increase in the number of altruistic living kidney donations. The United States Renal Data System 2015 report shows that 31% of all kidney donations are from live donors [4]. In addition to offering superior patient survival, kidney transplantation also results in a substantial economic savings as compared to treatment with dialysis [7].

A preemptive living donor transplant, before starting dialysis, has been shown to result in the best survival [8–10]. Now, 60 years after the performance of the first living donor transplant, more than 27,000 living donor kidney transplants are being performed annually worldwide [11, 12]. Yet, the availability of living kidney donors has stagnated or declined in the United States, Canada, Australia, New Zealand, Brazil and Europe, while exhibiting some growth in Japan and South Korea [13]. In spite of the excellent results of living donor kidney transplants in the United States, there has been a 10% decline from 6647 to 5989 in the years 2004 to 2013. Disproportionate decreases were seen in some subgroups, including males, related donors and donors aged less than 50 years [14–16].

Kidney donation is a decision with lifetime health implications. Given the relative safety of the surgical procedure itself, the central concern in relation to the removal of one kidney from a healthy individual is the long-term risk of progressive chronic kidney disease (CKD). However, there are long term studies in which the risk for kidney disease has remained small [17, 18]; the attributable risk for eventual kidney failure has been calculated at 27 per 10,000 (0.3%) at 15 years [17]. There is insufficient data available to determine lifetime attributable risk [19].

Barriers and challenges are faced by patients and their families in arranging kidney donation. In order to assist individuals with kidney failure to overcome these barriers in finding live kidney donors, transplant programs have established training seminars to teach potential transplant patients and their loved ones strategies to facilitate a living kidney donation [20]. Designated individuals are sometimes called “live donor champions”. Friends, family members or community members are trained by transplant centers to assist patients in finding altruistic kidney donors.

### History of kidney transplantation in Israel

Kidney transplantation has been available in Israel since the mid-1960s. The first two kidney transplants, one from a living related donor and the second from a deceased donor, were performed at approximately the same time at two different hospitals. Afterwards, kidney transplantation began to be performed in several hospitals throughout the country, but the overall numbers remained small.

**Fig. 1:**
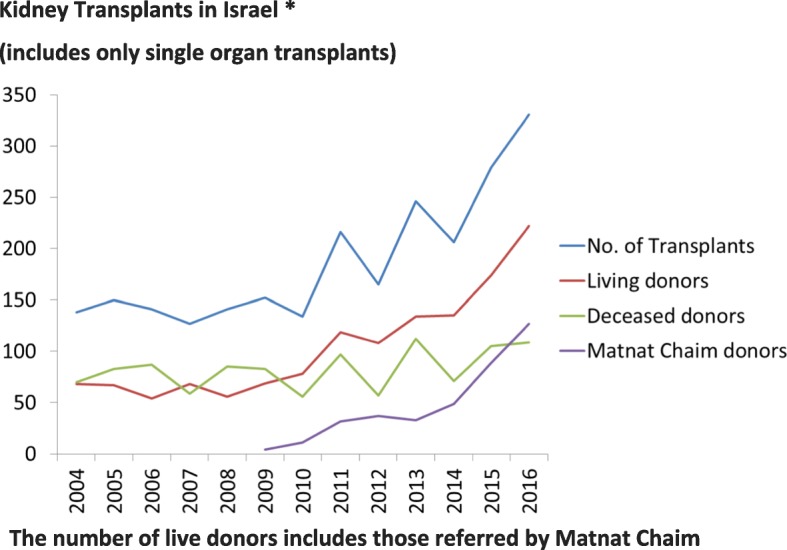
Kidney transplants in Israel, 2004–2016. This figure demonstrates the total number of kidney transplantations (blue), comparing the living donor transplants (red) deceased donor kidney transplants (green) to those facilitated by Matnat Chaim (purple). Starting in 2009, there has been a sharp increase in the total number of transplants, and this is mainly due to the increase in the number of live donor transplants, largely facilitated by Matnat Chaim. The number of transplants was obtained from the Israel Ministry of Health (http://www.health.gov.il/Subjects/Organ_transplant/transplant/Pages/default.aspx)

In 1994, the National Transplant Center was established as an Israel governmental organization. The objectives of the organization were to promote organ transplantation, to maintain a central list of potential transplant candidates, to select recipients as organs became available, according to established criteria, and to provide guidelines for various functions, such as selection of patients and collection of data. In spite of this, the number of kidney transplants remained less than 150 per year in the early 2000s (Fig. 1), a number insufficient to satisfy the needs of the relatively large number of potential recipients on the waiting list (Table 1). As a result of this, many Israelis with kidney disease travelled to other countries in order to undergo kidney transplantation using live or deceased donor kidneys. In some cases, this involved “organ trafficking”, which led to severe legislation and sanctions based on the adoption of Istanbul declaration principles (see below) [21].

**Table 1:** Number of individuals with late-stage chronic kidney disease (CKD) in Israel on the waiting list for kidney transplantation

Year	Number of patients on the waiting list for kidney transplantation
2004	453
2005	487
2006	490
2007	518
2008	540
2009	598
2010	690
2011	733
2012	729
2013	755
2014	762
2015	849
2016	843
2017	847

Source: The Ministry of Health, Israel (http://www.health.gov.il/Subjects/Organ_transplant/transplant/Pages/waiting_for_transplants.aspx)

The number of individuals with late-stage CKD in Israel on the waiting list for kidney transplantation has doubled in 13 years, but stabilized over the past three years. This could be largely due to the result of the efforts of the Matnat Chaim organization, established in 2009, which has facilitated an increasing number of Israeli kidney donors, especially over the past three years

In 2008, the Israeli Knesset enacted two laws [22–24]. The first defined brain death and provided the criteria for diagnosing brain death [25]. The second law, the transplantation law. defined the conditions for performing transplants using deceased and live donors in Israel [26, 27]. Furthermore, it made direct payments to donors and agents illegal [26]. Remuneration for loss of income was permitted and regulated. The regulations regarding the requirements for the use of organs from living donors were published. These included the completion of physical and psychological testing in one of the recognized transplant centers.

In addition, related donors are required to receive the approval of an independent committee in the hospital of the transplant center, whereas altruistic non-related donors are referred to an independent national committee for the approval of such donors. This committee requires not only the data from the transplant center, but presentation of data on the potential recipient and an independent psychological examination before confirming the particular donor-recipient pair. The performance of organ transplantation in foreign countries is allowed only in countries with strict regulations preventing organ trafficking. During the same year (2008), the Istanbul Declaration supported the prohibition of organ trafficking and transplant tourism [21]. This created a decrease in the number of Israeli patients obtaining kidney transplants in foreign countries, which resulted in an increase in the number of patients on the waiting list for kidney transplantation starting in 2008 (see Table 1).

### The attitude of Jewish religious leaders regarding organ transplantation

**Fig. 2:**
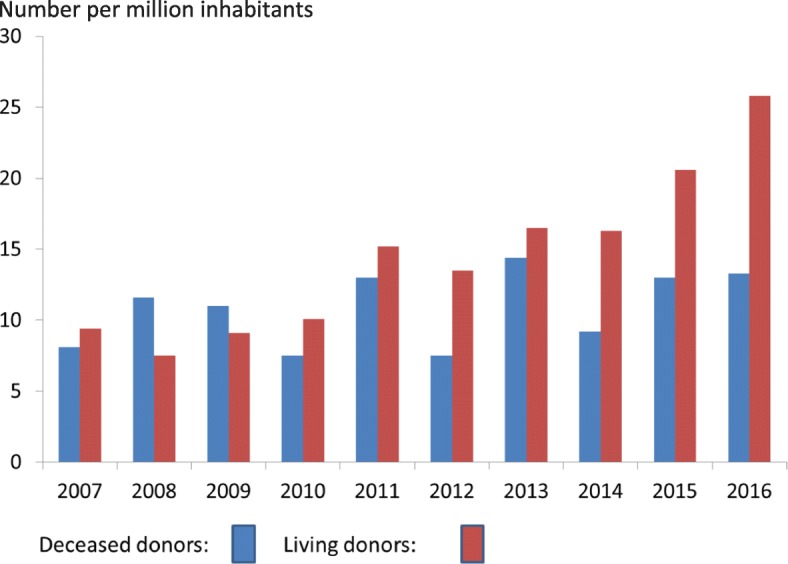
Kidney transplants in Israel by donor source from 2007 to 2016 (number of transplants per million population). The number of deceased donor transplants (blue) has increased by 64%, whereas the number of living donor transplants (red) has increased by 174%. The number of transplants was obtained from the Ministry of Health and the population figures from the National Bureau of Statistics

Historically, many, but not all, rabbinic authorities rejected the use of organs from deceased donors for transplantation [25]. The main objection was related to the definition of brain death, which is not considered acceptable to many rabbinical authorities, as opposed to cardiac death, which was universally accepted [28]. The harvesting of organs from a non-heart beating deceased donor is considered permissible by even the most stringent rabbinic authorities. Although in recent years there has been an increase in the percentage of families allowing the use of organs from deceased relatives, there has been no substantial increase in the actual number of deceased donors (Figs. 1 and 2). In contrast, while there may be some dispute regarding nonliving kidney donation, a virtual unanimity of opinion amongst all rabbinic authorities is to encourage, but not to require, living donor kidney donation [29].

### Matnat Chaim encourages live kidney donation among Orthodox Jews in Israel

The primary function of Matnat Chaim, a registered Israel non-profit organization (https://kilya.org.il/en/), is to encourage altruistic living kidney donation of Israeli citizens with an emphasis on donors from the Orthodox Jewish community. Matnat Chaim generates initial interest primarily through awareness-raising campaigns in the media, both traditional media, such as newspapers, magazines, radio and television, as well as new media, such as internet and social media sites, especially Facebook. The organization produces magazine supplements that disseminate stories describing the life and suffering of dialysis patients, as well as inspiring stories of kidney donors. As individuals, who have been exposed to a media campaign or who have heard about the organization, express an interest, Matnat Chaim invites these potential donors and their families to meetings where they may speak individually with staff and obtain medical information on kidney donation. The organization helps donors navigate the health system, and refers them to physicians with particular experience in advising kidney donors. The organization attempts to promote living related donation as a priority before matching the transplant candidate with an unrelated altruistic donor. It will not directly attempt to persuade a non-relative to donate a kidney. Only preliminary donor screens are provided via the organization with preliminary medical testing for kidney donation performed by the health maintenance organization (HMO) family physician. Potential donor and recipient pairs are then referred to a transplant center, where further examinations are performed and where it is decided whether the potential recipient and donor are compatible and medically suitable. In the case of an altruistic donor the donor-recipient pair is further interviewed by the National Committee for Altruistic Donors, which by law must take ultimate responsibility in approving each altruistic kidney donation to ensure inter alia that there is no commercial component.

Potential donors often request to donate to a person with specific characteristics, such as a child, a mother of small children, a non-smoker, or a member of a specific religious group. Although kidney donors are allowed to choose specific characteristics of the recipient, donors are not permitted to choose a specific recipient. Donor satisfaction and thereby the number of donors is increased by allowing donors this prerogative, the compelling benefit of which is considered by the National Committee for Altruistic Donors to outweigh potential harm. Relevant information is provided to the committee with consent of the participants.

The purpose of this paper is to describe the establishment of an organization for the facilitation of live kidney donations to Israel transplant centers wherein the donors are mostly from the Israeli Orthodox Jewish community. We hope to demonstrate how this organization affected the numbers of total kidney transplants by referring to data published by the Israel Ministry of Health.

## Methods

We performed a retrospective, record-based study of the 382 live kidney donor transplants facilitated by the Matnat Chaim Organization in Israel transplant centers (https://kilya.org.il/en/). Criteria for inclusion in the study were individuals referred by the organization for kidney transplantation after the establishment of the organization in February, 2009 until December 2016. These data were then compared to the total number of kidney transplantations performed from January 2004 to December 2016 (Israel Ministry of Health website). Data utilized in the figures and tables accessed from a link to the Ministry of Health (http://www.health.gov.il/Subjects/Organ_transplant/transplant/Pages/default.aspx) and the Israel National Bureau of Statistics http://www.CBS.gov.IL

The current study was conducted under the Ariel University Institutional Review Board. Since this was a retrospective record based study, no patient consent was required by the board.

## Results

**Fig. 3:**
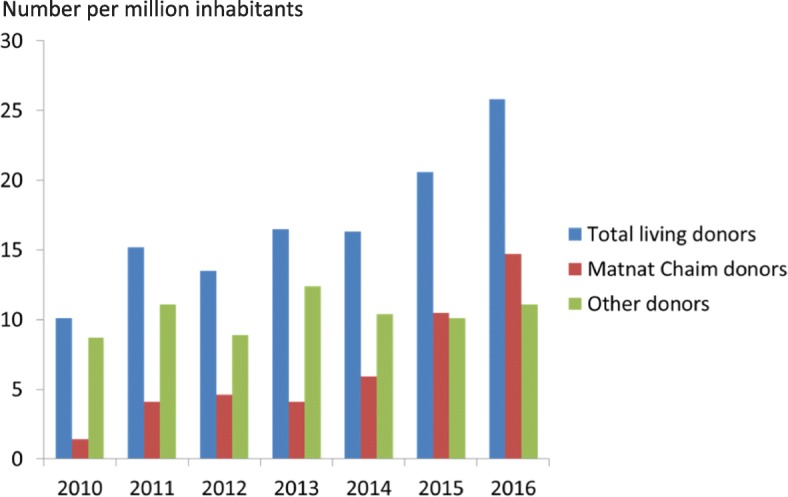
Total number of live donor transplants in Israel from 2010 to 2016 (number of transplants per million population). The number of live donor transplants in Israel is composed of those transplants facilitated by Matnat Chaim and other donors (i.e., those that were not facilitated by Matnat Chaim). This figure shows that the number of other live donor kidney transplants has remained stable at around 10 per million inhabitants over the past few years. The increase in total number of living donors per million inhabitants since 2013 appears due to the increase in donors facilitated by the Matnat Chaim organization. The “other donors” represents family members who donated not using Matnat Chaim as well as a small number of altruistic donors who went directly to a hospital or to the Israel Ministry of Health

The number of kidney transplants performed in Israel is depicted in Figs. 1, 2, 3. From 2004 until 2010 the yearly number of transplants ranged from 127 to 152 with approximately equal numbers of deceased kidney donors and live kidney donors. The numbers per million population are shown in Fig. 2. Following the passage of the Transplant law in 2008, the publication of criteria for reimbursement and the publication of preferential treatment for recipients whose family had previously donated organs or signed a donor card resulted in an increase in the number of deceased donors from a maximum of 87 until 2010 to 117 in 2013 and 115 in 2016 (Fig. 1). The number of live kidney donors increased from 78 in 2010 to 222 in 2016.

The number of kidney transplants per year per million population in Israel is presently 39. This places Israel in the group of countries performing 30 to 51 kidney transplants per million population, which includes 42% of countries worldwide [4]. The increase in the number of kidney transplants has resulted in a plateau in the number of patients on the transplant waiting list (Table 1). At the end of 2016, 847 individuals were listed on the Israel kidney transplant waiting list, representing 13% of the 6532 patients treated with renal replacement therapy.

The establishment of Matnat Chaim in 2009 was associated with a major increase in the number of live donor kidney transplants performed in Israel (Fig. 1). In 2011 only 27% of live donors were referred by Matnat Chaim whereas by 2015, 51% of all live kidney donors were referred by Matnat Chaim and by 2016 it had increased to 55% (Fig. 3). Matnat Chaim facilitated 4 live donor kidney transplants in 2009, 11 in 2010, 32 in 2011, 37 in 2012, 33 in 2013, 49 in 2014, 89 in 2015, 127 in 2016 and 112 live donor kidney transplants in 2017. In 2016, three of the 127 transplants facilitated by Matnat Chaim were altruistic donations from non-Israelis to citizens of other countries. The total number of live kidney donations facilitated by Matnat Chaim until the end of 2017 was 494. The mean age of these kidney donors was 41 years with the age range between 23 and 66 years; 73% were male and 27% were female.

Virtually all the donors referred by Matnat Chaim were altruistic. Specifically, of the 124 donors in Israel referred by Matnat Chaim in 2016, 111 (90%) were altruistic unrelated donations and only 13 (10%) were related to the recipient. In 2017, of 112 kidney donations, 98 (88%) were altruistic and 14 (22%) were related.

During the calendar year 2016, Matnat Chaim referred 324 potential kidney donors for evaluation. Of these, 185 were rejected for kidney donation by the family physician or the transplant center for medical reasons, including hematuria, proteinuria, hypertension, obesity (BMI > 30), low glomerular filtration rate, nephrolithiasis, cysts, and psychological problems. Furthermore, 12 potential kidney donors referred by Matnat Chaim that had been accepted for kidney donation by Israel transplant centers were rejected by the National Committee for Altruistic Donors without explanation.

## Discussion

Individuals with advanced kidney failure are understandably overwhelmed with the tasks associated with caring for themselves due to their severe physical illness, in addition to depression often associated with kidney disease [30]. Even with family support, many find the task of finding a suitable kidney donor to be unachievable. This has resulted in transplant programs training individuals to assist persons with late-stage kidney disease to organize kidney transplants. The emergence of a faith-based organization in Israel, whose purpose is to arrange living donor kidney transplantation, has resulted in a sharp increase in the number of live donor transplants, and represents a new development of advocacy for individuals with kidney disease that might garner wider activity. To the best of our knowledge, there are no previously published descriptions of similar live donor community organizations.

The impressive increase in numbers of live kidney donations, emanating mainly from the Orthodox Jewish community in Israel, resulted in a sharp increase in non-related altruistic donors and provided almost all these altruistic donors as shown in the number of kidney transplant donors for 2016. The success of this organization, has generated a debate regarding the ethics of directed live kidney donations by ethnicity. Ethicists voice conflicting opinions on this point. Some ethicists find the practice of directed live kidney donation acceptable [31] while others oppose the practice that kidney donors be allowed to choose the recipient [32]. These ethicists compare live kidney donation to deceased donation where the family is not allowed to choose the recipient and the recipient is chosen from a list, according to a medical priority setting algorithm [32]. Israeli law relegates these decisions to the National Committee for Evaluation of Living Donors who adjudicates the propriety of every nonrelated altruistic kidney donation. In turn, the committee policy has been to allow potential nonrelated donors to choose the characteristics of recipients in order to increase the number of live kidney donations for the benefit of all.

What features account for the success of this program? Persuasive elements of previous programs, such as those that utilize live-donor champions [20] or programs that advertise for kidney donors in print and social media [33] are also utilized by this organization. These include the use of “nonargumentative influence”, persuasive approaches to shaping behavior that does not attempt to use reason and rather utilizes emotional appeals, such as allowing the individual with kidney disease to tell his/her specific story [34]. The other very useful persuasive approach utilizes a “messenger effect”, the use of influential figures, such as peers or community leaders, techniques that give the kidney donation message greater weight in the eyes of the potential kidney donor [34]. The organization under the direction of Rabbi Heber, a charismatic and inspiring person, effectively utilizes this approach. The use of patient navigators by Matnat Chaim is another innovation that has been previously shown in a randomized, controlled trial to double the effective completion of the kidney transplant process by recipients [35].

Probably the most important and significant reason for the success of the program is the religious and spiritual importance that the kidney donors place on fulfilling the religious edict of saving another person’s life. This is epitomized by the Talmudic passage in the Babylonian Talmud, Tractate “Sanhedrin”, page 37a states that “he who saves one life is as if he has saved the entire world” [36].

One of the possible ethical problems of the activities of such a non-profit organization is the acceptance of donations from the public and from the families of potential recipients. It may be insinuated that such donations might possibly advance certain recipients. Accordingly, it is strongly recommended that fundraising and philanthropic support for such organizations must be led, handled, and processed by individuals not connected with the identification of potential kidney donors, or allocation to recipients. The limitations of this study are that the donor-recipient pairs have not been cross-identified to allow detailed description of their characteristics and a long-term study of the results of kidney transplantation on these individuals is not available. Recently, data regarding the low absolute risk for kidney failure among live donors, have been reported [37] and practice guidelines on the evaluation and care of live kidney donors have been published [38].

The Israel Transplant law was enacted in 2008 and Matnat Chaim began to facilitate live donor kidney transplants in 2009. The Israel Transplant law was associated with a dramatic reduction in the number of Israelis obtaining kidney transplants in countries outside Israel [39]. This legislation may have also played a coincident role in the outcome of increasing living kidney donations within Israel, thus facilitating the contribution of Matnat Chaim.

An additional benefit of the increase in the number of kidney transplants related to the activities of the Matnat Chaim organization is the estimated savings to the Israel Ministry of Health Budget. A recent investigation of kidney transplantation in Sweden showed a substantial savings due to kidney transplantation compared to treatment with dialysis [40]. Similarly, we estimate that the 494 individuals who received a kidney transplant due to the activities of Matnat Chaim will save the Israel healthcare budget an estimated $25 million in dialysis costs annually. This savings may be usefully contrasted with the reported Matnat Chaim annual operating budget of less than $1 million, raised totally from private funds.

Furthermore, studies are required to explore and to further understand emotional responses as an important motivator for living kidney donation. In particular, several of the present authors are examining demographic and psychological characteristics of past kidney donors for the purpose of developing a profile to identify potential future donors. It remains to be seen whether similar organizations of different religious and non-religious individuals can alter the donation rate in other countries.

## Conclusions

Matnat Chaim is an Israeli not-for-profit organization that has succeeded in facilitating 494 live kidney donations since its establishment in 2009 until the end of 2017, contributing to the doubling of the number of kidney transplantation procedures performed in Israel. The organization achieves its success by personalizing the kidney transplant process and addressing a potential donor’s concerns during the decision making process. Matnat Chaim is a unique kidney transplant success story that may serve as a model when applied in other religious and non-religious transplant community organizations worldwide.
